# The Antimicrobial Resistance of *Candida*: A 5-Year Retrospective Analysis at a Tertiary Hospital in Jordan

**DOI:** 10.3390/jof11020087

**Published:** 2025-01-23

**Authors:** Anas H. Abu-Humaidan, Areen Alshdaifat, Dima Awajan, Mohammad Abu-Hmidan, Abeer Alshdifat, Hanan Hasan, Fatima M. Ahmad, Nader Alaridah, Amal Irshaid, Dina Yamin

**Affiliations:** 1 Department of Pathology, Microbiology and Forensic Medicine, School of Medicine, The University of Jordan, Amman 11942, Jordan; a.abuhumaidan@ju.edu.jo (A.H.A.-H.); ary0194543@ju.edu.jo (A.A.); mabuhemidan@gmail.com (M.A.-H.); f_ahmad@ju.edu.jo (F.M.A.); n.alaridah@ju.edu.jo (N.A.); amal.irshaid17@gmail.com (A.I.); 2 Department of Clinical Pharmacy and Therapeutics, Applied Science Private University, Amman 11931, Jordan; dimaawajan@gmail.com; 3 Faculty of Medicine, Yarmouk University, Irbid 21163, Jordan; abeershdefat.40@gmail.com; 4 Department of Medical Laboratory Sciences, School of Science, The University of Jordan, Amman 11942, Jordan; hna8190719@ju.edu.jo; 5 Microbiology & Immunology Lab, University of Jordan Hospital, Amman 11942, Jordan

**Keywords:** *Candida*, *albicans*, *tropicalis*, *Nakaseomyces glabratus*, *Pichia kudriavzevii*, registry, antifungal resistance, hospital-acquired infections candidiasis, surveillance

## Abstract

*Candida* infections are a global health concern, increasingly complicated by rising antimicrobial resistance (AMR). This study analyzed the prevalence and AMR patterns of circulating *Candida* species in Amman, Jordan, using electronic records from a tertiary teaching hospital’s microbiology lab (from 2017 to 2022). Complete records of *Candida* isolates (n = 2673) were assessed by sample type, species, and AMR. Among positive blood samples, *C. albicans* accounted for the majority (38.7%), followed by *C. tropicalis* (19.0%), *C. parapsilosis* (18.3%), *Nakaseomyces glabratus* (14.6%), and *Pichia kudriavzevii* (9.5%). Non-*albicans* species demonstrated higher resistance to Caspofungin, notably *P. kudriavzevii* (23.1%), *N. glabratus* (30.0%), and *C. parapsilosis* (32.0%), compared to *C. albicans* (1.9%). In high vaginal swabs, *C. albicans* was most prevalent (63.7%), with *N. glabratus* also notable (28.6%); Fluconazole resistance in *C. albicans* remained low (2.0%). Across all pooled isolates, AMR was similar between inpatients and outpatients, except for Micafungin, where inpatient resistance was significantly higher. In conclusion, non-*albicans* species predominated in blood infections and demonstrated pronounced AMR. Micafungin resistance was notably higher among inpatients. Variations in *Candida* species and AMR by sample type suggest that aggregating samples in registry studies may obscure critical patterns.

## 1. Introduction

Fungal infections are a growing global burden, and *Candida* species are the most isolated species in invasive fungal infections, both in the community and in hospitals [1], with a high mortality rate and treatment cost [2]. Such infections pose a bigger threat to immunocompromised, organ transplant, and cancer patients [3]. Additionally, fungal infections were a noticeable factor in death during the COVID-19 pandemic [4]. Continuous surveillance of *Candida* infections is required to note the changes in the prevailing species and their antimicrobial resistance (AMR).

In October 2022, the World Health Organization (WHO) launched the Fungal Priority Pathogens List (FPPL), a pivotal initiative designed to classify fungal pathogens based on their threat levels. The critical priority group consists of *Candida auris* and *Candida albicans*. The high priority group includes *Nakaseomyces glabratus* (formerly *Candida glabrata*), *Candida parapsilosis*, and *Candida tropicalis*. Finally, the medium priority group encompasses pathogens such as *Pichia kudriavzevii* (previously *Candida krusei*). Despite these updated classifications, infections caused by these fungi will continue to be collectively referred to as “candidiasis” for practical purposes [5,6].

Nearly 90% of *Candida* (*C.*) infections are caused by the five main species: *C. albicans*, *N. glabratus*, *C. tropicalis*, *C. parapsilosis*, and *P. kudriavzevii* [7]. A review of the epidemiology of invasive fungal infections in Arab countries indicated that few had published reports [8], with the majority of studies originating from Lebanon, Saudi Arabia, and several other Gulf states [9,10]. Those studies focused on *Candida* isolated from the bloodstream and showed that *C. albicans* was the most common isolate with rates ranging from 22.3 to 60.0%, but indicated that non-*albicans* species were on the rise [11]. Studies from Jordan and Egypt used population data, mainly from the World Health Organization (WHO), to estimate the burden of invasive fungal diseases and concluded that around (2%) of the Egyptian population is affected by fungal infections [12]. Likewise, in Jordan, serious fungal infections were estimated to affect nearly 119,000 patients (1.9%), excluding cutaneous fungal infections [13]. Such studies based on population data could estimate the burden of invasive fungal disease but not the causative *Candida* species.

AMR is a growing global problem that is more prevalent in low- and middle-income countries, partly due to the misuse of antimicrobials and the prevalence of communicable diseases [14]. In Jordan, self-medication with antimicrobials was recently reported to be high [15]. Regionally, a study conducted in Lebanon and Saudi Arabia showed that less than 62% of the tested *Candida* isolates were susceptible to Fluconazole [9]. In the US, a large population study from several centers over a 4-year period found that 7% of all *Candida* blood samples were resistant to Fluconazole, and 1.6% were resistant to echinocandins [16]. The growing AMR was shown to be more substantial in some non-*albicans* species, such as *P. kudriavzevii*, *C. auris*, and *C. parapsilosis*, some of which are described as multi-drug resistant (MDR) [17]. Previous studies on pathogenic *Candida* species in Jordan targeted susceptible populations such as cancer patients [18] or diabetic patients [19]. However, the burden of candidiasis is expected to increase in Jordan and globally as predisposing conditions that lead to immunosuppression, such as increased life expectancy and the availability of cancer treatments, become more common [20].

Given the paucity of data from the region and the emerging MDR *Candida* species, this study aimed to describe *Candida* isolates in terms of the infections they cause, the causative species, their AMR, and whether AMR differed between inpatients and outpatients, using data that spanned 5 years from a teaching tertiary hospital in the capital Amman, Jordan.

## 2. Materials and Methods

### 2.1. Study Design and Data Collection

This study was an observational epidemiological study based on data collected from the electronic Microbiology Lab records at Jordan University Hospital (JUH) covering the period of June 2017 to June 2022; to ensure data accuracy and minimize potential errors, the electronic records were cross-referenced with clinical charts and laboratory results whenever possible. JUH is a tertiary teaching hospital in Jordan’s capital, Amman, with roughly 100,000 tests handled in the Microbiology Lab yearly. The records included the type and date of the sample requested for culture, the isolated pathogen, and its antifungal sensitivity pattern. Patients’ age and gender, as well as their inpatient vs. outpatient status, were registered.

### 2.2. Pathogen Identification and Antimicrobial Susceptibility Testing

The isolation and presumptive identification of clinically relevant *Candida* species was conducted through the examination of colony color and morphology on chromogenic agar media (CHROMID^®^ *Candida*, bioMérieux, Marcy-l’Étoile, France) after 48 h of incubation in aerobic conditions at 35 to 37 °C. For uncharacterized colonies and the confirmation of the presumptive identification of isolated *Candida* species [21], the automated system (VITEK^®^ 2, bioMérieux, Marcy-l’Étoile, France) with the identification card (YST ID card, VITEK^®^ 2, bioMérieux, Marcy-l’Étoile, France) was used. This system has become a standard practice in many government hospital laboratories in Jordan due to its cost-effectiveness, accessibility, and ease of implementation in routine diagnostics. Also, a germ tube test was sometimes used to rapidly distinguish *C. albicans*, where a suspected white colony was incubated in bovine serum at 37 °C for 2 to 3 h to detect germ tube formation microscopically [22]. These methods, although not without limitations, are widely employed in government hospital laboratories due to their practicality, cost-effectiveness, and ease of integration into routine diagnostic workflows.

The automated and standardized testing card (YS08 AST card, VITEK^®^ 2, bioMérieux, Marcy-l’Étoile, France) was used to test the antimicrobial susceptibility testing of the isolated *Candida* species against Caspofungin, Fluconazole, Micafungin, Voriconazole, Amphotericin B, and Flucytosine. The Clinical and Laboratory Standards Institute (CLSI) breakpoints outlined in the Performance Standards for Antimicrobial Susceptibility Testing (M60) were used [23]. Where clinical breakpoints were not established, such as for Amphotericin B, epidemiological cutoff values (ECVs) from CLSI (M59) were applied to aid in the interpretation of susceptibility results [24], and if the new breakpoints were not proposed for a combination (for example *N. glabratus*—Voriconazole), the older breakpoint was used. Similarly, sample collection, handling, and the reporting of results followed updated CLSI guidelines (see Supplementary Table S1 for details).

### 2.3. Data Analysis

The raw data were extracted from the electronic Microbiology Lab records as Microsoft Excel spreadsheets and cleaned to focus solely on *Candida* infections. A total of 3073 records containing pure *Candida* isolates were found. Records lacking sensitivity results and records with ambiguous or missing sample sources were excluded, leaving 2673 records.

A preliminary analysis indicated that the five main species of *C. albicans*, *C. tropicalis*, *P. kudriavzevii*, *N. glabratus*, and *C. parapsilosis* were found in 2649 or 99.1% of all complete *Candida* records and were the focus of analysis. The analysis included blood samples from inpatients, high vaginal swabs (HVS) from outpatients, sputum and bronchoalveolar lavage (BAL) samples from inpatients, and urine samples from both patient groups. Other sample types were excluded mainly due to low testing numbers.

Fisher’s exact test was applied to assess differences in *C. albicans* AMR across different sample types and to assess differences in AMR between inpatients and outpatients. The test was two-tailed, and a *p*-value ≤ 0.05 was considered statistically significant.

## 3. Results

### 3.1. Candida Species Isolated from Inpatient Blood Samples and Their Antifungal Resistance

**Figure 1:**
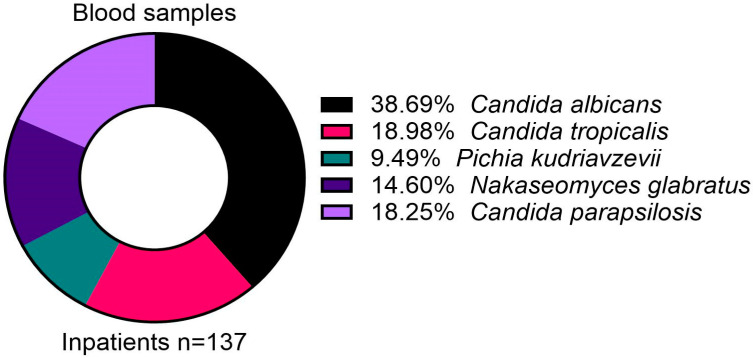
Distribution of isolated *Candida* species from blood samples in inpatients.

One hundred and thirty-nine blood samples were positive for *Candida*, of which 137 were attributed to five main species (Figure 1). While *C. albicans* (38.7%) formed most of the positive blood samples, the non-*albicans* species, including *C. tropicalis* (19.0%), *P. kudriavzevii* (9.5%), *N. glabratus* (14.6%), and *C. parapsilosis* (18.3%) collectively formed over 60% of samples (Figure 1).

Non-*albicans* species displayed higher resistance to echinocandins; notably, the resistance to Caspofungin in *C. tropicalis* (7.7%), *P. kudriavzevii* (23.1%), *N. glabratus* (30.0%), and *C. parapsilosis* (32.0%) was higher than *C. albicans* (1.9%). Meanwhile, resistance to Micafungin was demonstrated in *N. glabratus* (6.3%) and *C. parapsilosis* (5.9%) only. Fluconazole resistance, on the other hand, was high in *C. tropicalis* (9.1%) and low in *C. albicans* (1.9%), with no resistant *C. parapsilosis* isolates. Interestingly, no resistant isolates to Voriconazole were found (Table 1).

To further investigate antifungal resistance in blood isolates, the percentage of resistant isolates was analyzed over the 5-year study period. The data revealed two main peaks of increased resistance, occurring in the second half of 2018 and the first half of 2021 (Supplementary Figure S1).

Additionally, patient demographics, including age and sex, were compared between those with resistant and nonresistant blood isolates to explore potential associations with AMR for each antifungal. Although the small number of resistant isolates limited statistical analysis, the median age of patients with resistant blood isolates was generally higher than those with nonresistant isolates across all tested antifungals (Supplementary Table S2). The distribution of male and female patients varied by antifungal. A higher percentage of males was observed in the Caspofungin and Micafungin-resistant groups, while females were more represented in the Fluconazole-resistant group (Supplementary Table S2).

### 3.2. Candida Species Isolated from High Vaginal Swabs (HVS) and Their Antifungal Resistance

**Figure 2:**
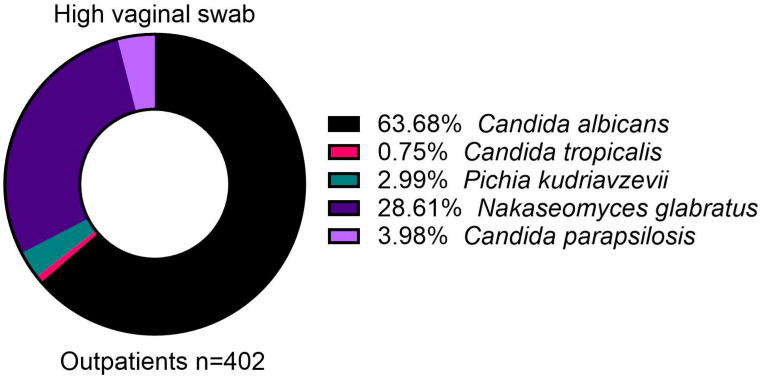
Distribution of isolated *Candida* species from high vaginal swabs samples in outpatients.

A total of 404 high vaginal swabs from outpatients were positive for *Candida*, from which 402 were attributed to the five main species (Figure 2). *Candida albicans* formed most of the positive samples (63.7%), with a notable minority of *N. glabratus* (28.6%) (Figure 2). Importantly, the resistance rate to Fluconazole was low in *C. albicans* (2.0%) (Table 2). While *C. parapsilosis* formed only 4.0% of samples, it had an unusual resistance rate to Fluconazole, with 15 out of the 16 isolates (93.8%) showing resistance (Table 2).

### 3.3. Candida Species Isolated from Sputum and Bronchoalveolar Lavage (BAL) Fluid and Their Antifungal Resistance

**Figure 3:**
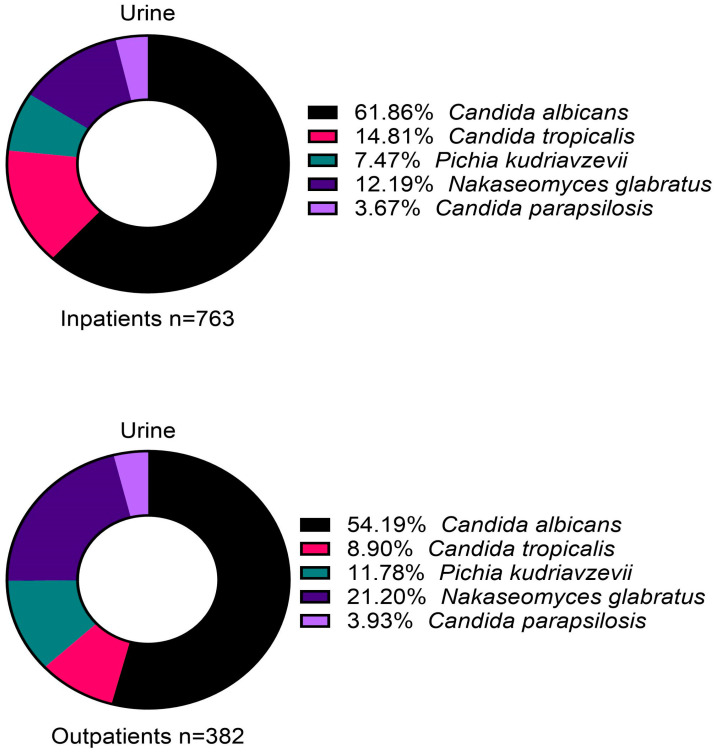
The distribution of isolated *Candida* species from sputum and bronchoalveolar lavage (BAL) samples in inpatients.

A total of 697 sputum and BAL fluid samples from inpatients tested positive for *Candida*, all of which were attributed to the five main species (Figure 3). *Candida albicans* was the most common (83.1%), while *C. tropicalis* formed 7.0% of isolates.

Resistance to Caspofungin was notable in the non-*albicans* species, namely, *P. kudriavzevii* (21.7%), *N. glabratus* (13.2%), and *C. parapsilosis* (37.5%) (Table 3). Flucytosine resistance rates were highest in *P. kudriavzevii* (95.7%) (Table 3), whereas they were significantly lower in *C. albicans* (5.0%) and *N. glabratus* (4.1%) (Table 3). Like HVS isolates, Fluconazole resistance was especially high in *C. parapsilosis* (14.3%) (Table 3).

### 3.4. Candida Species Isolated from Urine Samples and Their Antifungal Resistance

**Figure 4:**
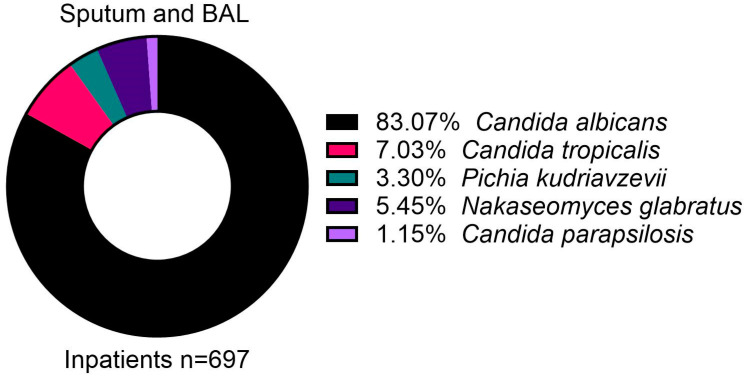
Distribution of isolated *Candida* species from urine samples in inpatients (upper panel) and outpatients (lower panel).

Here, 770 urine samples from inpatients were positive for *Candida*, of which 763 were attributed to the five main species. Similarly, 388 urine samples from outpatients were positive for *Candida*, of which 382 were attributed to the same five species (Figure 4). There were slight differences in the isolated species between inpatients and outpatients, with *C. albicans* forming most isolates both in inpatients (61.9%) and outpatients (54.2%), but *N. glabratus* formed a larger minority in outpatients (21.2%) compared to inpatients (12.2%) (Figure 4).

Like our findings in HVS and sputum/BAL samples, *P. kudriavzevii* exhibited the highest resistance rates to Flucytosine in inpatient and outpatient urine samples (94.7% and 100.0%, respectively) (Table 4 and Table 5). In contrast, Fluconazole showed resistance rates of 40% and 14.3% in outpatients and inpatients, respectively, while Caspofungin exhibited rates of 0% and 37.5%, respectively (Table 4 and Table 5).

### 3.5. Comparing the Antifungal Resistance of C. albicans Isolated from Different Sites

**Figure 5:**
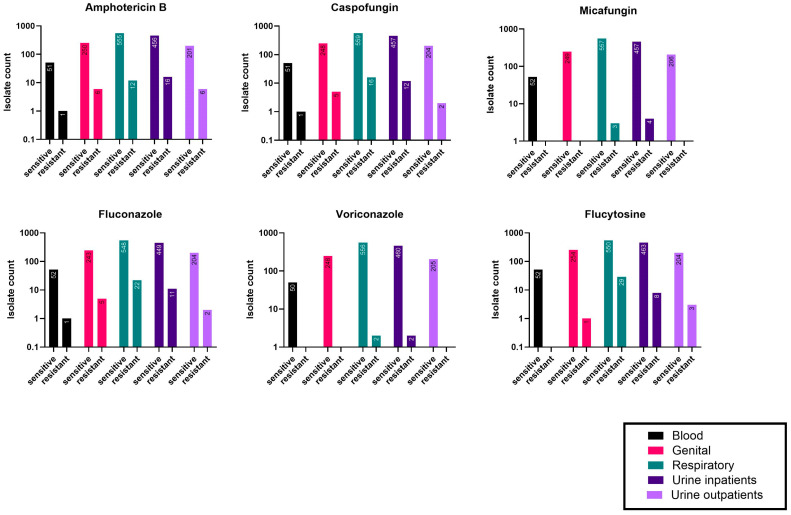
Resistance of *C. albicans* isolated from different sample types. The number of resistant isolates and nonresistant isolates (including sensitive and intermediate) is displayed in the bars for each tested antifungal. Different colors represent different sample types (explained in the lower left corner). Fisher’s exact test was used to compare the number of resistant isolates for each antibiotic in each sample type, and no significant differences were observed except for Flucytosine (*p* = 0.0003). Genital tract samples were high vaginal swabs (HVS), and respiratory samples were sputum and bronchoalveolar lavage (BAL) fluid samples.

Antimicrobial resistance in *C. albicans* isolates from different sample types (blood, HVS, urine, and BAL fluid) was compared for each antifungal. Fisher’s exact test revealed no significant differences, except for Flucytosine, where resistance appeared to be sample-dependent (p = 0.0003). Notably, *C. albicans* isolates from respiratory samples showed the highest AMR rate at 5.0%, compared to lower rates in other sample types (Figure 5).

### 3.6. Comparing the Antifungal Resistance Among Inpatient and Outpatient Candida Isolates

To determine if AMR in *Candida* species differed between inpatients and outpatients, all *Candida* isolates from inpatients (n = 1972) and outpatients (n = 1101) were pooled, regardless of species or sample source. Resistance rates were similar for most antifungals across both groups, except for Micafungin, which showed significantly higher resistance among inpatients than outpatients (1.4% vs. 0.4%, respectively, *p* = 0.0108) (Table 6).

## 4. Discussion

This study analyzed *Candida* isolates in terms of species and AMR patterns using data collected over five years (between 2017 and 2022) from electronic records of a tertiary teaching hospital in Amman, Jordan. Out of over 3000 positive *Candida* results, 2673 were included after excluding incomplete records. The five main *Candida* species accounted for 2649 cases (99.1%). This dataset represents one of the largest collections in Jordan in recent years, offering an updated perspective on the rising AMR trends in the region and globally, and enabling a comparison of the AMR of *Candida* isolated from inpatients and outpatients.

The retrieval of *Candida* species from blood samples is considered an invasive fungal disease episode according to the recent Consensus Definitions of Invasive Fungal Disease [25]. This study found that 139 blood samples were positive for *Candida*, with predominance in non-*albicans* species, a common finding in most regions of the world. A recent study from China on invasive fungal infections in hospitalized patients between 2013 and 2018, which used the aforementioned definition, indicated that the most commonly isolated fungal species were *C. parapsilosis* in 34.8% of 509 patients, followed by *C. guilliermondii* (26.7%), and *C. albicans* (18.5%) [26]. In an 18-year study on *Candida* species blood isolates, a shift towards the isolation of non-*albicans Candida* has been observed. Recently, *C. parapsilosis* was noted to be the most predominant species, contributing to 29.2% (343/1175) of candidemia, followed by *C. albicans* (20.1%) [27]. Conversely, a study in Italy that investigated 270 episodes of candidemia from 2010 to 2014 showed a similar distribution of pathogens retrieved from blood samples to our study, with *C. albicans*, *C. parapsilosis,* and *C. tropicalis* forming 54%, 23%, and 10%, respectively, among all isolates [28].

A retrospective study from a closer geographical location that investigated candidemia cases in Saudi Arabia from 2002 to 2009 showed that non-*albicans* were found in 65.9% of the total 258 positive blood cultures for *Candida*, compared to around 60% of blood samples observed in our study that were caused by non-*albicans* species. The reported non-*albicans Candida* species in that study compared to our study were *C. tropicalis* (15.5% vs. 19.0%, respectively), *C. parapsilosis* (11.9% vs. 18.3%, respectively), *N. glabratus* (9.1% vs. 14.6%, respectively), and *P. kudriavzevii* (4.0% vs. 9.5%, respectively) [29]. Interestingly, another study conducted on 2075 positive blood isolates in Kuwait from 2006 to 2017 indicated that *C. albicans* (37.22%) was closely followed by *C. parapsilosis* (34.66%), while *P. kudriavzevii* formed less than 2% of isolates [30].

In general, the above studies indicate that the *Candida* species isolated from blood samples were mainly *C. albicans,* followed by *C. parapsilosis*, *C. tropicalis*, or *N. glabratus,* with a high degree of agreement between studies on the four commonest species. However, a difference stands out about *P. kudriavzevii*, which was isolated from a higher percentage of blood samples in our study (9.5%) compared to all the aforementioned studies. *Pichia kudriavzevii* was described to vary in its temporal and geographical distribution in a large study conducted by the ARTEMIS DISK Antifungal Surveillance Program from 2001 to 2005, which included 124 medical centers from different countries, and reported the highest rate of *P. kudriavzevii* isolation from blood samples in the Czech republic as 7.6%, which is still lower than the rate in our study [31].

Echinocandins such as Caspofungin and Micafungin are some of the most recent additions to antifungals and are a first-line treatment in selected patients with invasive fungal infections [32], but resistance to echinocandins has been increasing over the past two decades, especially in *N. glabratus*, with rates ranging from less than 2% to over 10% in some institutions in the US and Europe [33]. Results of our study showed that Caspofungin and Micafungin resistance in blood isolates was highest in *N. glabratus* (30.0% and 6.3%, respectively) and *C. parapsilosis* (32.0% and 5.9%, respectively), a noteworthy finding given the high mortality associated with echinocandin treatment failure as one recent report from Switzerland described [34]. Interestingly, both species are classified as high priority in the new WHO classification due to their increasing resistance rates and their role in causing severe infections.

In addition to the high rate of resistance to echinocandins, *P. kudriavzevii* was practically invulnerable to Flucytosine as resistance rates were around 92% in inpatient isolates, similar to a previous large-scale study in which only 8% of 254 bloodstream isolates of *P. kudriavzevii* were susceptible to Flucytosine [31]. Resistance to Amphotericin B was noticeable as well in *P. kudriavzevii* isolates (7.7%) but was comparable to studies showing increased resistance to this antifungal [31].

While *Candida* species can be retrieved from HVS from asymptomatic women, *Candida* retrieved from HVS in a diagnostic setting is often the causative agent in vulvovaginal infections [35]; thus, most of the isolated *Candida* species in this study could be considered pathogenic. The prevalence of *Candida* species from HVS was similar to that of global studies, which indicate the predominance of *C. albicans*. However, *N. glabratus* formed a higher minority in this study (28.6%) than most reports, where it represents less than 20% [36,37]. The resistance to Fluconazole, which is the first-line treatment for such infections, was infrequent in *C. albicans* since only 2% of the 256 isolates were found to be resistant, which supports Fluconazole use as the first-line treatment [38]. However, the widespread practice of using empirical treatments may result in incomplete or inappropriate regimens, complicating the effective management of *Candida* infections. The availability of over-the-counter antifungal vaginal treatments poses challenges like misdiagnosis and drug resistance. Vaginal symptoms may stem from conditions beyond fungal infections, such as bacterial vaginosis or sexually transmitted infections. Without medical advice, improper use of antifungals may delay care and worsen outcomes, highlighting the need for standardized protocols and professional oversight [39].

The higher AMR exhibited by the non-*albicans* species *P. kudriavzeveii* and *N. glabratus* compared to *C. albicans* in this study and previous literature [40,41], could be attributed to the genetic divergence between these genera. Unlike *C. albicans*, species within the genera *Pichia* and *Nakaseomyces* often exhibit intrinsic resistance to Fluconazole, primarily due to the upregulation of efflux pumps, some of which are absent in *C. albicans* [42]. Similarly, intrinsic resistance to Flucytosine, exceeding 90% for *P. kudriavzeveii* in this study, is rare among *Candida* species but is a shared trait of many *Pichia* species [43]. These genetic differences highlight the importance of accurate species classification in understanding AMR patterns and guiding effective antifungal therapy.

It is difficult to differentiate contamination or colonization from infection when *Candida* is isolated from urine because no diagnostic test would command a conclusive result [44]. *Candida* forms part of the skin microbiota and can contaminate urine cultures [45], and at the same time, *C. albicans* and *N. glabratus* could colonize the external part of the urethra in female patients. This colonization could explain the notable minority of *N. glabratus* we found in outpatients. In any case, *C. albicans* formed most isolates in both inpatients and outpatients, while *C. tropicalis* was the second most common species in inpatients, where predisposing factors for candiduria are usually present, such as prolonged antibiotic use, catheterization, and immunosuppression. Studies investigating community-acquired and hospital-acquired candiduria have had varying results concerning differences in the species distribution between the two populations [46]. The AMR analysis of those species to Fluconazole, often used as a first-line treatment in urinary tract infections, indicated that less than 3% of both FPPL critical and high priority groups (*C. albicans* and *C. tropicalis*) were resistant. Similarly, those species, in addition to *N. glabratus*, had more than 98% isolates sensitive to Flucytosine, which could be used as a second-line treatment.

Given the colonization of most mucosal surfaces in the body with various *Candida* species, it is also difficult to assess the significance of a positive *Candida* isolate from sputum or BAL samples; nevertheless, some studies indicate that isolating *Candida* species in patients with severe lung disease was associated with worst outcomes [47]. This study indicated that *C. albicans* was the dominant species, with non-*albicans* forming less than 20% of samples, and the resistance of those isolates to the tested antifungals was lower than 4%, except for Flucytosine where the resistance rate was 5%.

An analysis of *C. albicans* AMR by sample type showed no significant difference for five of the six antifungals tested. However, Flucytosine resistance varied, being under 2% for all sample types except respiratory samples, where it reached 5%. The required antifungal concentration to effectively kill fungal pathogens varies by tissue, potentially contributing to the emergence of resistant isolates due to suboptimal drug penetration into specific tissues or secretions, such as sputum and BAL fluid [48]. Alternatively, this may result from a statistical anomaly or misidentification of other fungal pathogens such as *C. albicans*, but in any case, *C. albicans* isolated from respiratory samples is often regarded as colonization rather than an active pathogen [49].

The significant difference in Micafungin resistance rates between inpatient (1.4%) and outpatient (0.4%) populations may reflect differences in patient health status, exposure to healthcare settings, and prior antifungal use. Inpatients often have more complex comorbidities and may receive multiple courses of antibiotics or antifungals, which can contribute to the development of resistant strains, highlighting the need for tailored antifungal strategies based on patient demographics and clinical settings. As resistance patterns evolve, continuous evaluation of treatment protocols and emerging antifungal therapies will be essential in managing invasive candidiasis effectively [50].

While the use of electronic records for epidemiological studies such as this one has limitations, it is a crucial data source, not only in resource-limited settings where alternative data sources may not be feasible but also in developed countries, with many initiatives in the field of antimicrobial stewardship based on pooling data from the electronic records of several centers [51]. The relatively large sample size in this study, combined with validation measures proposed for studies based on electronic records [52], minimized the likelihood of significant errors in our data. As an example of external validation, the distribution of *Candida* species across various sample types in this study was consistent with epidemiological findings from similar populations in the region; likewise, AMR observed in our study aligned with previous regional and global research. As for internal validation, we examined the congruence of available variables in the data; for example, we confirmed that all high vaginal swabs were from female patients, and we cross-checked inpatient versus outpatient status, confirming that blood samples were predominantly from inpatients, except for those from the emergency department, and we removed any sample with incomplete information such as missing the sample source. Finally, we randomly examined records from inpatients with sensitive and resistant isolates to confirm, through progress or discharge notes, the credibility of the culture and sensitivity results.

It should be noted that this study used the Vitek-2 system in both identification and AMR testing, and while it is not considered a reference method, several studies have shown it has excellent agreement with the CLSI or European Committee on Antimicrobial Susceptibility Testing (EUCAST) reference methods, both in terms of identification and susceptibility testing [53,54]. However, a drawback in this method was the exclusion of the following combinations of sensitivity testing: *N. glabratus*—Fluconazole and *P. kudriavzevii*—Fluconazole, since those combinations were not part of the Food and Drug Administration (FDA) indications for the use of the AST-YS08 card [55]. Additionally, given our study’s design, it was impossible to conclude with certainty whether the isolated *Candida* species from certain sample types was pathogenic.

Finally, variability in the sensitivity testing results of Caspofungin could be associated with false resistance reports, especially in the case of *N. glabratus* [56]. Unfortunately, due to resource limitations, FKS gene sequencing to confirm resistance was not performed. This constraint may have resulted in an overestimation of Caspofungin resistance in this study.

## 5. Conclusions

The distribution of *Candida* species in Jordan largely aligned with regional and global trends, though there was a higher prevalence of non-*albicans* species, especially *P. kudriavzevii,* in blood and HVS samples. Additionally, non-*albicans* species exhibited relatively high resistance to Caspofungin, which should inform candidemia management strategies. AMR was similar between inpatients and outpatients, except for Micafungin, where inpatient resistance was significantly higher. This study also highlighted that *Candida* species and AMR profiles can vary by sample type, suggesting that combining different sample types may obscure meaningful patterns in registry studies.

## Figures and Tables

**Table 1:** Resistance among *Candida* isolates from blood samples in inpatients ^1,2^.

	Species	*C. albicans* (n = 53)	*C. tropicalis* (n = 26)	*P. kudriavzevii* (n = 13)	*N. glabratus* (n = 20)	*C. parapsilosis* (n = 25)
Antifungal						
Amphotericin B		1.9%	4.0%	7.7%	0.0%	8.3%
Caspofungin		1.9%	7.7%	23.1%	30.0%	32.0%
Micafungin		0.0%	0.0%	0.0%	6.3%	5.9%
Fluconazole		1.9%	9.1%	NA	NA	0.0%
Voriconazole		0.0%	0.0%	0.0%	0.0%	0.0%
Flucytosine		0.0%	4.0%	92.3%	0.0%	0.0%

^1^ Percentages indicate the proportion of resistant isolates out of the total number of isolates for each species. ^2^ NA; Not available since those combinations were not reported using the antimicrobial susceptibility testing card YS08.

**Table 2:** Resistance among *Candida* isolates from high vaginal swabs (HVS) in outpatients ^1,2^.

	Species	*C. albicans* (n = 256)	*C. tropicalis* (n = 3)	*P. kudriavzevii* (n = 12)	*N. glabratus* (n = 115)	*C. parapsilosis* (n = 16)
Antifungal						
Amphotericin B		2.3%	0.0%	0.0%	1.8%	0.0%
Caspofungin		2.0%	0.0%	0.0%	20.4%	6.3%
Micafungin		0.0%	0.0%	0.0%	0.0%	0.0%
Fluconazole		2.0%	33.3%	NA	NA	93.8%
Voriconazole		0.0%	0.0%	0.0%	0.0%	0.0%
Flucytosine		0.4%	0.0%	100.0%	1.7%	0.0%

^1^ Percentages indicate the proportion of resistant isolates out of the total number of isolates for each species. ^2^ NA; Not available since those combinations were not reported using the antimicrobial susceptibility testing card YS08.

**Table 3:** Resistance among *Candida* isolates from sputum and bronchoalveolar lavage samples in inpatients ^1,2^.

	Species	*C. albicans* (n = 579)	*C. tropicalis* (n = 49)	*P. kudriavzevii* (n = 23)	*N. glabratus* (n = 38)	*C. parapsilosis* (n = 8)
Antifungal						
Amphotericin B		2.1%	0.0%	4.3%	7.9%	0.0%
Caspofungin		2.8%	2.0%	21.7%	13.2%	37.5%
Micafungin		0.5%	0.0%	10.0%	4.5%	0.0%
Fluconazole		3.9%	4.2%	NA	NA	14.3%
Voriconazole		0.4%	0.0%	0.0%	0.0%	0.0%
Flucytosine		5.0%	4.1%	95.7%	0.0%	0.0%

^1^ Percentages indicate the proportion of resistant isolates out of the total number of isolates for each species. ^2^ NA; Not available since those combinations were not reported using the antimicrobial susceptibility testing card YS08.

**Table 4:** Resistance among *Candida* isolates from urine samples in inpatients ^1,2^.

	Species	*C. albicans* (n = 472)	*C. tropicalis* (n = 113)	*P. kudriavzevii* (n = 57)	*N. glabratus* (n = 93)	*C. parapsilosis* (n = 28)
Antifungal						
Amphotericin B		3.4%	0.9%	3.6%	1.1%	0.0%
Caspofungin		2.6%	6.3%	10.9%	19.5%	14.8%
Micafungin		0.9%	2.8%	4.2%	0.0%	0.0%
Fluconazole		2.4%	2.8%	NA	NA	24.0%
Voriconazole		0.4%	0.9%	0.0%	1.1%	0.0%
Flucytosine		1.7%	0.0%	94.7%	2.2%	0.0%

^1^ Percentages indicate the proportion of resistant isolates out of the total number of isolates for each species. ^2^ NA; Not available since those combinations were not reported using the antimicrobial susceptibility testing card YS08.

**Table 5:** Resistance among *Candida* isolates from urine samples in outpatients ^1,2^.

	Species	*C. albicans* (n = 207)	*C. tropicalis* (n = 34)	*P. kudriavzevii* (n = 45)	*N. glabratus* (n = 81)	*C. parapsilosis* (n = 15)
Antifungal						
Amphotericin B		2.9%	5.9%	6.7%	9.9%	0.0%
Caspofungin		1.0%	9.1%	0.0%	26.9%	0.0%
Micafungin		0.0%	0.0%	0.0%	3.2%	0.0%
Fluconazole		1.5%	0.0%	NA	NA	40.0%
Voriconazole		0.0%	0.0%	0.0%	1.3%	0.0%
Flucytosine		1.4%	0.0%	100.0%	6.2%	0.0%

^1^ Percentages indicate the proportion of resistant isolates out of the total number of isolates for each species. ^2^ NA; Not available since those combinations were not reported using the antimicrobial susceptibility testing card YS08.

**Table 6:** Resistance among all *Candida* isolates from inpatients vs outpatients ^1,2^.

Antifungal	Inpatients (n = 1972)	Outpatients (n = 1101)	*p*-Value
Amphotericin B	3.0%	3.4%	0.59
Caspofungin	7.4%	7.5%	0.94
Micafungin	1.4%	0.4%	0.01
Fluconazole	4.6%	5.5%	0.33
Voriconazole	0.6%	1.2%	0.08
Flucytosine	8.7%	9.0%	0.79

^1^ Percentages indicate the proportion of resistant isolates out of total isolates regardless of species and sample source. ^2^ A two-tailed Fisher’s exact test was used to obtain an exact *p*-value.

## Data Availability

The data that support the findings of this study are available upon request from the corresponding author.

## Supplementary Materials

The following supporting information can be downloaded at: https://www.mdpi.com/article/10.3390/jof11020087/s1. Figure S1: The percentage of resistant isolates from blood samples to each antifungal across the study period. Each year was divided into two halves; H1 January–June, and H2: July–December; Table S1: Clinical Minimum Inhibitory Concentration (MIC) Breakpoints for Selected *Candida* Species Based on CLSI Documents M60 (1st Edition) and M59 (2nd Edition); Table S2: Age and sex of patients with resistant vs. non-resistant isolates from blood samples. Reference [57] is cited in the Supplementary Materials.
