# Supplementary material for: The Antimicrobial Resistance of Candida: A 5-Year Retrospective Analysis at a Tertiary Hospital in Jordan
Source: J Fungi (Basel). 2025 Jan 23;11(2):87. doi: 10.3390/jof11020087 (PMC11857050; doi:10.3390/jof11020087)
Supplement: Supplementary file 1 [file jof-11-00087-s001.zip › jof-3364062-supplementary.pdf]

Table S1. Clinical Minimum Inhibitory Concentration (MIC) Breakpoints for Selected *Candida* Species Based on CLSI Documents M60 (1st Edition) and M59 (2nd Edition).

		CLSI ECVs and Breakpoints (µg/mL)				
Species	Drug	S (≤)	I	SDD	R (≥)	ECV
Candida albicans	Amphotericin B	—	—	—	—	2
	Micafungin	0.25	0.5	—	1	0.03
	Fluconazole	2	—	4	8	0.5
	Caspofungin	0.25	0.5	—	1	—
	Flucytosine	—	—	—	—	0.5*
	Voriconazole	0.12	0.25–0.5	—	1	0.03
*Nakaseomyces glabratus (*formerly *Candida glabrata)*	Amphotericin B	—	—	—	—	2
	Micafungin	0.06	0.12	—	0.25	0.03
	Fluconazole	—	—	≤32	64	8
	Caspofungin	0.12	0.25	—	0.5	—
	Flucytosine	—	—	—	—	0.5*
	Voriconazole	—	—	—	—	0.25
Pichia kudriavzevii (formerly *Candida krusei*)	Amphotericin B	—	—	—	—	2
	Micafungin	0.25	0.5	—	1	0.25
	Fluconazole	—	—	—	—	—
	Caspofungin	0.25	0.5	—	1	—
	Flucytosine	—	—	—	—	32*
	Voriconazole	0.5	1	—	2	0.5
Candida parapsilosis	Amphotericin B	—	—	—	—	2
	Micafungin	2	4	—	8	2
	Fluconazole	2	—	4	8	2
	Caspofungin	2	4	—	8	—
	Flucytosine	—	—	—	—	0.5*
	Voriconazole	0.12	0.25–0.5	—	1	—
Candida tropicalis	Amphotericin B	—	—	—	—	2
	Micafungin	0.25	0.5	—	1	0.06
	Fluconazole	2	—	4	8	1
	Caspofungin	0.25	0.5	—	1	—
	Flucytosine	—	—	—	—	0.5*
	Voriconazole	0.12	0.25-0.5	—	1	0.12

Abbreviations: CLSI, Clinical and Laboratory Standards Institute; S, susceptible; SDD, susceptible, dose-dependent; R, resistant; ECV, epidemiological cutoff value.

* For Flucytosine ECVs referenced from (1)

1. Pfaller M, Espinel-Ingroff A, Canton E, Castanheira M, Cuenca-Estrella M, Diekema D, et al. Wild-type MIC distributions and epidemiological cutoff values for amphotericin B, flucytosine, and itraconazole and Candida spp. as determined by CLSI broth microdilution. Journal of clinical microbiology. 2012;50(6):2040-6.

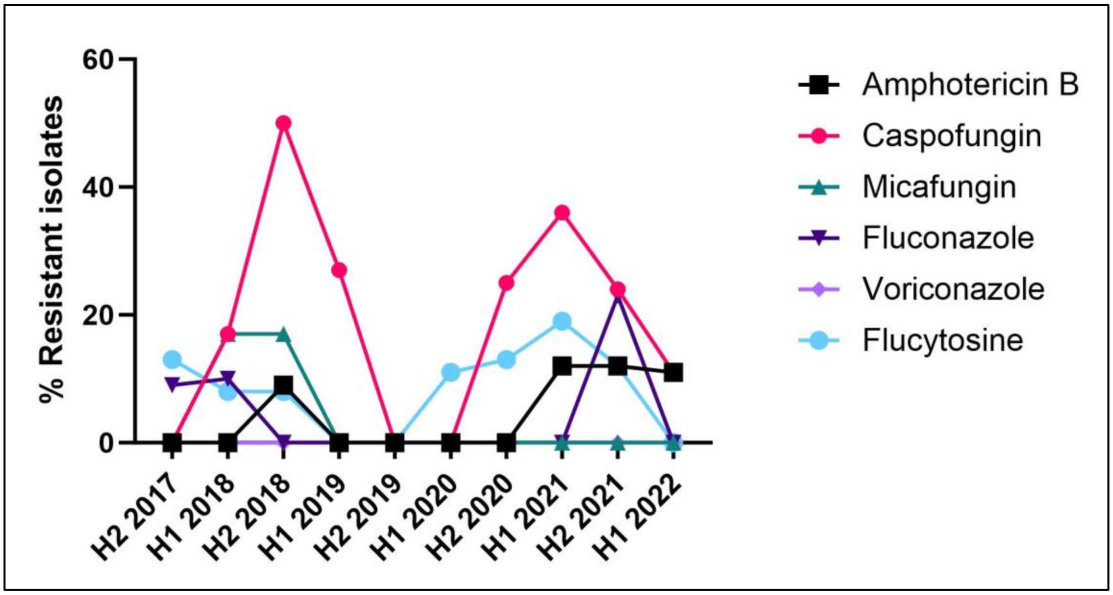

Figure S1. The percentage of resistant isolates from blood samples to each antifungal across the study period. Each year was divided into two halves; H1 January- June, and H2: July- December.

Table S2. Age and sex of patients with resistant vs. non-resistant isolates from blood samples.

Antifungal	Median age in years (95% CI)		Female %	
	Resistant	Non-resistant	Resistant	Non-resistant
Amphotericin B	55 (9-66)	43 (38-48)	57.1%	46.7%
Caspofungin	58 (39-61)	42 (37-47)	23.8%	51.6%
Micafungin	62 (27-48)	42 (36-47)	0.0%	50.4%
Fluconazole	64 (28-87)	39 (35-45)	100.0%	46.9%
Voriconazole [1]	N/A	44 (38-48)	N/A	46.6%
Flucytosine	69 (40-70)	42 (37-47)	64.3%	45.7%

[1] No blood isolates were resistant to Voriconazole during the study period.
